# Glycemic Control in Diabetic Patients Receiving a Diabetes-Specific Nutritional Enteral Formula: A Case Series in Home Care Settings

**DOI:** 10.3390/nu16162602

**Published:** 2024-08-07

**Authors:** Paola Pantanetti, Giovanni Cangelosi, Marco Sguanci, Sara Morales Palomares, Cuc Thi Thu Nguyen, Giulio Morresi, Stefano Mancin, Fabio Petrelli

**Affiliations:** 1 Unit of Diabetology, Asur Marche–Area Vasta 4, 63900 Fermo, Italy; dr.paolapantanetti@gmail.com (P.P.); giovanni.cangelosi@virgilio.it (G.C.); 2 A.O. Polyclinic San Martino Hospital, Largo R. Benzi 10, 16132 Genova, Italy; sguancim@gmail.com; 3 Department of Pharmacy, Health and Nutritional Sciences (DFSSN), University of Calabria, 87036 Rende, Italy; sara.morales@unical.it; 4 Department of Pharmaceutical Administration and Economics, Hanoi University of Pharmacy, Hanoi 100000, Vietnam; cucnguyen.pharm@gmail.com; 5 Politecnica University of Marche, 60121 Ancona, Italy; giulio.morresi@gmail.com; 6 IRCCS Humanitas Research Hospital, 20089 Rozzano, Italy; stefano.mancin@humanitas.it; 7 School of Pharmacy, Polo Medicina Sperimentale e Sanità Pubblica “Stefania Scuri”, Via Madonna delle Carceri 9, 62032 Camerino, Italy

**Keywords:** diabetic patients, enteral nutrition, glycemic control, glycemic variability, home care

## Abstract

Background and Aim: In patients with Diabetes Mellitus (DM), Enteral Nutrition (EN) is associated with less hyperglycemia and lower insulin requirements compared to Parenteral Nutrition (PN). The primary aim of this study was to assess changes in glycemic control (GC) in DM patients on EN therapy. The secondary objectives included evaluating the impact of the specialized formula on various clinical parameters and the tolerability of the nutritional formula by monitoring potential gastrointestinal side effects. Methods: We report a case series on the effects of a Diabetes-Specific Formula (DSF) on GC, lipid profile (LP), and renal and hepatic function in a DM cohort receiving EN support. Results: Twenty-two DM subjects with total dysphagia (thirteen men, nine women) on continuous EN were observed. The use of a DSF in EN was associated with an improvement in glycemic indices across all patients studied, leading to a reduction in average insulin demand. No hospitalizations were reported during the study period. Conclusion: The study demonstrated that the use of DSFs in a multi-dimensional home care management setting can improve glycemic control, reduce glycemic variability and insulin need, and positively impact the lipid profile of the DM cohort. The metabolic improvements were supported by the clinical outcomes observed.

## 1. Introduction

In patients with Diabetes Mellitus (DM), Enteral Nutrition (EN) is associated with reductions in hyperglycemia and insulin requirements compared to Parenteral Nutrition (PN) treatment [1]. Additionally, EN is recommended over PN due to its increased safety profile [2]. However, managing enteral feeding in patients with DM can be challenging. Despite adherence to current guidelines for glycemic control (GC), only 40% of DM patients achieve the target glycemic range [3]. Hyperglycemia is a frequent issue in hospitalized subjects receiving EN, increasing the risk of hospital-related complications and mortality, regardless of prior DM diagnosis [4,5].

Elevated blood glucose (BG) levels pose a hazard in both critically and non-critically ill patients in hospital settings. Contributing factors include pre-existing DM, undiagnosed DM, prediabetes, stress hyperglycemia (SH), and glucocorticoid use [6,7]. Several factors can interfere with the GC and metabolic profile of patients receiving EN, such as delayed gastric emptying and impaired glucose absorption [8,9,10]. Continuous intestinal exposure to glucose influences the secretion and action of incretins, including gastric inhibitory polypeptide (GIP) and glucagon-like peptide-1 (GLP-1), which contribute to a reduction in glycaemia, often observed in patients, regardless of the history of DM, undergoing EN [8,11,12]. However, hyperglycemia is observed in 30% of patients undergoing EN [13,14], underlining the need for the careful management of glycemic levels to mitigate the associated risks. Several studies on GC and other clinical parameters during enteral tube feeding have been conducted in critically ill patients [15,16,17,18], but none have focused on the home care setting. Only one randomized controlled trial (RCT) has evaluated the role of insulin in patients receiving EN support, comparing Sliding-Scale Regular Insulin (SSRI) alone or in combination with glargine. Although no significant difference in GC was observed between the groups, about half of the SSRI group required additional Neutral Protamine Hagedorn (NPH) insulin twice daily due to persistent hyperglycemia, with over 50% of these patients having no prior DM diagnosis [19]. A few retrospective studies have reported varying degrees of GC and hypoglycemia rates in patients treated with different insulin regimens, including once-daily glargine, twice-daily NPH, every six hours NPH, biphasic insulin, and SSRI [20,21]. These findings highlight the need for the careful monitoring of EN effects in individuals with DM, using formulas that minimize glycemic and metabolic disturbances and optimize lipid profiles, including Total Cholesterol (TC), Triglycerides (TG), Low-Density Lipoprotein (LDL), and High-Density Lipoprotein (HDL). The efficacy of diabetes-specific nutritional therapy for GC and complication progression in diabetes patients is supported by the European Society for Clinical Nutrition and Metabolism (ESPEN) guidelines [22], which endorse the use of Diabetes-Specific Formulas (DSFs). An expert consensus on EN for adult patients with DM or hyperglycemia also recommends DSFs containing low-glycemic index carbohydrates and a moderate to high percentage of monounsaturated fatty acids to improve metabolic control during EN [23]. The GARIN working group similarly recommends DSFs for managing non-critical patients with DM, SH, and artificial nutrition (AN) [24]. DSFs typically feature lower carbohydrate content compared to standard formulas (SFs), higher amounts of complex carbohydrates that are slowly digestible to prevent blood glucose spikes, modified carbohydrates such as maltodextrin, starch, isomaltulose, fructose, and sucromalt, and higher proportions of unsaturated fats, especially monounsaturated fatty acids [25]. They also contain higher fiber content compared to SFs [26]. Studies have demonstrated that DSFs are associated with reduced postprandial blood glucose and insulin levels, mean blood glucose, glycemic variability (GV), short-acting insulin requirements, and changes in glycated hemoglobin (HbA1c) [27].

### Study Aim

The primary aim of this study was to assess changes in GC in DM patients on EN therapy. The secondary objectives included evaluating the impact of the specialized formula on various clinical parameters and the tolerability of the nutritional formula by monitoring potential gastrointestinal side effects. 

## 2. Materials and Methods

### 2.1. Study Design and Ethical Consideration

The study was a case series design and reported in accordance with the CARE guidelines [28]. It adhered to the principles outlined in the Helsinki Declaration. All participants were informed about the study’s objectives, and written consent was obtained in compliance with all privacy regulations (Art. 13 EU Regulation 679/2016). The data were processed anonymously. Ethical approval was granted by the Ethics Committee (approval number n.2135/2022).

### 2.2. Setting, Participants, and Criteria

This study included a cohort of adult patients with DM on insulin therapy and receiving EN. Participants were enrolled through convenience sampling from a home care program managed by the Azienda Sanitaria Unica Regionale (ASUR) Area Vasta 4 of Fermo, Italy, between January and June 2023. Eligibility was based on their participation in the home care program, which delivers healthcare services to individuals unable to independently visit healthcare facilities. All DM patients on insulin therapy and receiving EN within the home care program during the specified period were included in the study. Exclusion criteria comprised hospital admission for any reason and death during the observation period.

### 2.3. Assessment and Procedures 

All patients were assessed at baseline and at 3 and 6 months after starting treatment with Glucerna^®^, a specialized EN formula designed for patients with diabetes [29]. The composition of the DSF used in this study is detailed in Table 1. The caloric and protein goals for each patient were determined based on a comprehensive dietary assessment conducted prior to the initiation of enteral nutrition. This assessment was performed by a dietitian and a nutritionist. The goals varied across the sample, reflecting differences in precise caloric and protein intake requirements, which were tailored according to individual patient needs. Factors influencing these requirements included the presence of comorbid conditions and other patient-specific variables. As a result, the enteral nutrition regimens were customized to optimize the nutritional support for each patient. The assessment of the tolerance to the nutritional formula was based on data recorded in the medical records. Any potential complications attributable to gastrointestinal factors, such as nausea, diarrhea, abdominal pain, or other related symptoms, were documented as alterations in tolerance to the DSF.

The following clinical and biochemical parameters were analyzed: Body Mass Index (BMI), blood pressure (BP), blood glucose (BG), lipid profile (LP), albumin, complete blood count, serum electrolytes, indices of liver and kidney function, and uric acid as an additional cardiovascular risk factor in diabetic patients [30].

Additionally, HbA1c levels were measured and GV was estimated using Kovatchev’s indices. Data collection was meticulous and systematic. Information was extracted from a shared electronic folder maintained collaboratively by the Diabetology and primary care centers of the referral district. This centralized data repository ensured the accuracy and consistency of the information collected. The role of the home nurse was pivotal in the success of this study. Acting as a crucial link between the hospital and the community, the home nurse managed each participant comprehensively. Responsibilities included overseeing self-care practices, administering prescribed therapies, preventing pressure ulcers, and monitoring for diabetes-related complications. This comprehensive approach ensured that each participant received personalized and continuous care throughout the study period. Overall, these procedures facilitated a comprehensive and longitudinal assessment of the impact of a specialized EN support formula tailored for patients with diabetes on their health outcomes.

### 2.4. Data Analysis

Various clinical and biochemical parameters were systematically measured. The collected data were processed and analyzed using JASP 0.18.1, a free statistic software (https://jasp-stats.org/; accessed on 3 February 2024). Descriptive statistics were calculated for continuous variables, while categorical variables were summarized using frequencies and percentages. Comparative analyses were conducted to evaluate changes in the clinical and biochemical parameters over time at 3 and 6 months. Correlation analyses were performed to explore potential relationships between the various parameters; GV was estimated using Kovatchev’s indices.

## 3. Results

### 3.1. Characteristics of the Participants

The study included twenty-two patients with DM, consisting of thirteen men and nine women, with a mean age of 72 ± 8 years and a mean BMI of 21.6 ± 3.4 kg/m^2^. All participants exhibited total dysphagia and received continuous EN support over 24 h. Nutritional support was administered either via Percutaneous Endoscopic Gastrostomy (PEG) (*n* = 12) or a Nasogastric Tube (NGT) (*n* = 10). Patients were provided with a specialized EN support formula which is specifically designed for individuals with DM or SH.

### 3.2. Insulin Requirements

**Figure 1:**
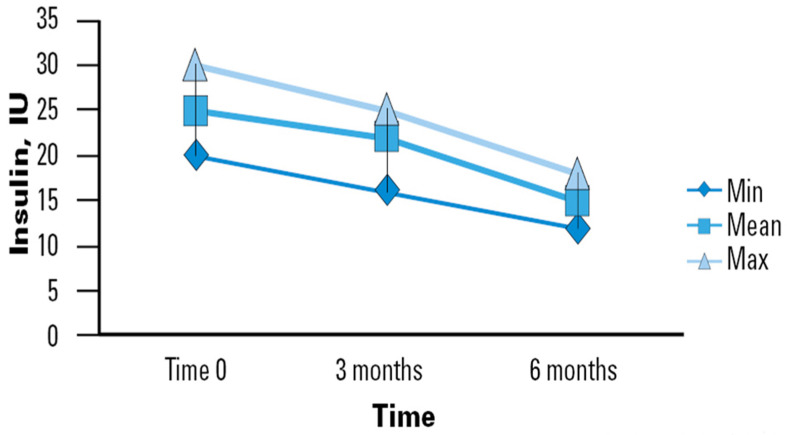
Time course of insulin requirements in diabetic patients receiving DSF. Legend. DSF = Diabetes-Specific Formula; IU = International Unit; Min = Minimum; Max = Maximum.

The use of a specialized EN support formula was associated with mean improvement in BG levels in the sample studied, which translates to a lower insulin therapy demand (Figure 1). In our study, the initial average daily insulin dose upon enrollment was observed to be 25 International Units (IU), with a range typically between 20 and 25 IU. By the second time point (3 months), the average daily dose decreased slightly to 24 IU, ranging from 17 to 26 IU. By the final observation period (6 months), there was a notable reduction in the average daily dose to 17 IU, with a range between 13 and 19 IU. This significant decrease from 25 to 17 IU (Δ − 8) demonstrates the effectiveness of the enteral nutrition intervention over both short and medium terms. Our study highlights the progressive stabilization of insulin requirements in older adults who regularly consumed the studied nutritional intervention. Although not all reductions between measurement points reached statistical significance, a clear trend toward decreased insulin requirements over time was observed. This trend suggests that the improvement in GC may be progressive and stabilize in the long term. Such observations could indicate the stabilization of glucose metabolism in elderly patients with a prolonged use of the specific nutritional formula.

### 3.3. Impact of DSF on Clinical Variables

Baseline measurements were compared with data collected at the 6-month follow-up to assess changes indicative of treatment efficacy. A total of eight parameters were systematically analyzed: BG, HbA1c, Creatinine, uric acid, TC, HDL, LDL, and TG. The results revealed significant alterations across several metrics following treatment initiation. Notable findings included a mean decrease in BG levels from 145 mg/dL at baseline to 128 mg/dL at 6 months accompanied by a reduction in HbA1c levels from 9.8% to 7.7%. Additionally, markers of renal function such as Creatinine and uric acid exhibited favorable trends, with mean changes of 0.08 mg/dL and 2.2 mg/dL, respectively.

LP demonstrated improvements, exemplified by a decrease in TC levels from 288 mg/dL to 240 mg/dL, an increase in HDL cholesterol from 31 mg/dL to 42 mg/dL, and a significant decrease in LDL cholesterol from 187 mg/dL to 120 mg/dL. TG levels also decreased substantially from 300 mg/dL to 160 mg/dL.

The observed improvements underscored the efficacy of the EN support formula in modulating key clinical parameters associated with diabetes management and cardiovascular health. These trends indicate that the intervention with the specific nutritional formula has beneficial effects not only on glycemic control but also on LP and renal function. Although some changes may not be statistically significant, the observation of positive trends across multiple clinical parameters suggests an overall improvement in the metabolic health of patients (Table 2).

### 3.4. Glycemic Variability

**Figure 2:**
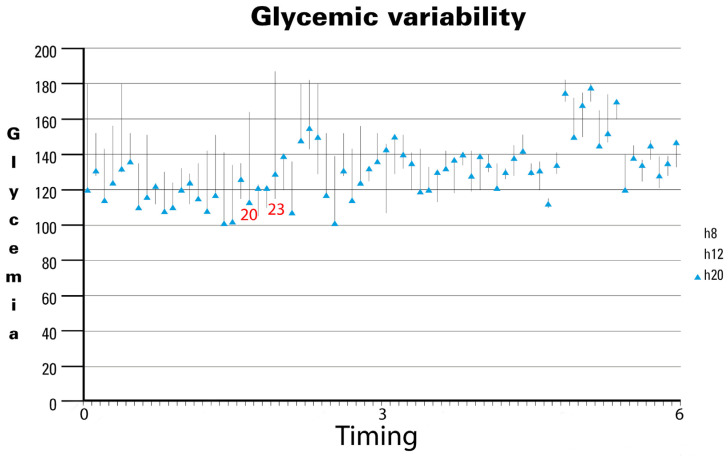
Glycemic variability in diabetic patients receiving DSF. Legend. DSF = Diabetes-Specific Formula.

In the last analysis, GV showed a significant reduction over time (Figure 2). Specifically, analyzing the 70 most significant observations during the study period revealed notable trends. Initially, GV exhibited wide fluctuations, as seen in observation 20 (approximately 2 months after initiating the specialized EN support formula), ranging from 116 mg/dL to 161 mg/dL, and observation 23, which ranged from 118 mg/dL to 182 mg/dL. However, as the study progressed, GV stabilized noticeably, with minimal variability observed in the later stages. Our findings underscore the long-term stabilization of GV, despite average BG levels remaining between 140 mg/dL and 160 mg/dL in the fifth month. Notably, no hospitalizations were reported throughout the study period, likely reflecting the effectiveness of the nursing care provided. The observed reduction in GV over time suggests that the specific nutritional formula contributes to maintaining glycemic stability in patients. Although mean BG levels did not drop below 140 mg/dL, the decreased fluctuations indicate more stable and predictable glycemic control, which is crucial for long-term diabetes management and the prevention of complications.

### 3.5. Tolerability of the DSF 

In our study, the tolerability of the DSF was thoroughly assessed in all participating patients. All patients demonstrated adequate tolerance to the nutritional mixture, and, throughout the observation period, no gastrointestinal tract side effects were reported. Specifically, there were no instances of diarrhea, abdominal pain, or nausea, which are common concerns with dietary interventions.

## 4. Discussion

In our study, the use of DSFs tended to lower Hgb A1c, BG, and insulin requirements, with the caveat of a consistent carbohydrate intake throughout the observation period. This observation is consistent with the existing literature demonstrating the benefits of DSFs compared to SFs for individuals on insulin therapy and enteral feeding. Multiple studies have shown that DSFs, containing approximately 36% less total carbohydrate than SFs, significantly decrease postprandial BG responses up to four-fold [28,29,30,31,32]. The carbohydrate composition of DSFs plays a crucial role in these outcomes. DSFs are formulated with a blend of low-glycemic index, slow-digesting carbohydrates, along with added prebiotic fibers such as fructooligosaccharides (FOS) [29,30]. Clinical evidence supports the hypothesis that fructose, when consumed in controlled amounts throughout the day, reduces postprandial BG responses [33]. Additional carbohydrate sources in DSFs include resistant maltodextrins, isomaltulose, and sucromalt. These disaccharides are more resistant to digestive enzymes, resulting in a slower and more prolonged release of glucose into the bloodstream [33]. This gradual glucose release reduces the need for both endogenous and exogenous insulin, subsequently lowering the risk of hypoglycemia [29]. Furthermore, the GV, a critical parameter for monitoring glucose control, measures the extent, frequency, and duration of 24 h BG fluctuations. Alongside HbA1c, GV has become a widely recognized indicator of glucose control [34,35]. GV is particularly significant due to its correlation with oxidative stress and potential vascular endothelial damage, which can lead to diabetic complications [36]. Recent research has linked high GV with unfavorable clinical outcomes in DM [37].

Our study’s findings on the stabilization of GV support these observations. Over the study period, we observed a significant reduction in GV, indicating improved glucose control among participants using the DSFs. This stabilization is crucial for reducing the risk of diabetes-related complications and improving overall clinical outcomes. Additionally, the absence of hospitalizations during the study period highlights the effectiveness of the specialized EN support formula combined with nursing care.

Consequently, optimizing EN procedures in patients with diabetes is a significant challenge for clinicians and the multidisciplinary team. DSFs have been shown to reduce the risk of hyperglycemia, eliminate the risk of significant hypoglycemia, and ensure a higher percentage of BG values between 70 and 180 mg/dL, effectively reducing GV compared to SFs [28]. These benefits can help reduce the risk of acute complications associated with EN. This study demonstrated that the use of specialized formulas significantly reduces postprandial glycemic response, mean BG levels, and insulin requirements. These findings suggest that DSFs are beneficial for long-term glucose control in DM patients on insulin therapy receiving EN. Additionally, the higher GLP-1 levels associated with these specialized formulas may further facilitate the reducing of postprandial BG levels, indicating their superiority in managing diabetes compared to SFs or MUFA-rich low-carbohydrate formulas [32]. Moreover, our study corroborates these findings, demonstrating that the specialized formula can minimize GV in diabetic patients, leading to an average reduction in BG and HbA1c levels. These improvements are accompanied by enhanced renal function and a more favorable LP. Specifically, the reduction in various biochemical parameters such as UA, TC, LDL, and TG would contribute to a decreased cardiovascular risk. This effect would be particularly significant when these reductions are associated with an increase in HDL levels in diabetic patients, as demonstrated in previous studies [30,38].

The absence of hospital admissions during the study period underscores the strategic importance of DSFs for patients with diabetes. One of the key strengths of our case series study is its duration, with monitoring at 3 and 6 months after the initiation of EN with DSFs. Previous studies evaluating these parameters have generally been of a much shorter duration [39,40]. Our study confirms that the beneficial effects of the specialized formula on GV are maintained over the long term, providing sustained improvements in diabetes management.

The findings of our study are consistent with previously published research, demonstrating that the use of DSFs provides better control of BG levels compared to SFs. This metabolic improvement, coupled with modern health management strategies, supports enhanced clinical outcomes and a reduction in hospitalizations for DM patients on EN therapy or standard care [41,42,43].

### Limitations and Future Perspectives

Several limitations to this study need to be considered. Firstly, its retrospective case series design and small sample size, characteristic of a pilot study, along with the lack of preliminary clinical analysis and stratification of the enrolled population, may have introduced unknown confounding factors that could have impacted the results. Furthermore, it was not possible to estimate the exact nutritional intake, particularly carbohydrate intake, at each observation point. Initially, nutritional intake was calculated, but it was subsequently not considered due to the high risk of bias stemming from the home management of nutrition by patients and caregivers, as well as the varying doses of supplementation tailored to individual nutritional needs. Additionally, there was difficulty in defining the type of insulin used by each patient, as different formulations with varying effects might have been administered, further complicating the analysis. We also only calculated the means for each parameter at baseline and after 6 months due to the retrospective nature and limited sample size, which prevented us from performing detailed statistical analyses to infer the significance of the observed changes. Given these limitations, additional studies with larger sample sizes and more rigorous research methodologies are recommended. This case series serves as a precursor to a prospective cohort study with a larger sample size, aiming to confirm these preliminary findings and produce more robust results.

## 5. Conclusions

The results of this pilot study suggest that for patients with DM on continuous EN via PEG or NGT, the use of DSFs with high protein content and slow-releasing carbohydrates in a multi-dimensional care setting may significantly improve metabolic control. Specifically, our findings indicate improvements in mean BG levels and GC, as evidenced by a significant reduction in HbA1c, LP, TC, minimized GV, and the prevention of urgent hospital admissions. However, the retrospective and observational nature of this study, along with the small sample size, highlight the need for further research. RCTs with larger sample sizes are essential to validate these preliminary findings and to establish more robust evidence for the efficacy of DSFs in improving clinical outcomes in DM patients. Such studies should also explore the benefits of DSFs in a collaborative home care setting to confirm their impact and facilitate their integration into standard care practices.

## Figures and Tables

**Table 1:** Composition of DSF for diabetic patients on EN therapy.

Kcal: 120 kcal/100 mL
Carb: 32.24% (10.08 g/100 mL)
Protein: 19.98% (6 g/100 mL)
Fats: 44.95% (6 g/100 mL)
Fiber: 2.83% [1.70 g/100 mL of which short chain Fructooligosaccharides (FOS) 1 g/100 mL]
Vitamins and Minerals: Vitamin A, Vitamin D_3_, Vitamin E, Vitamin K_1_, Vitamin C, Folic Acid, Vitamin B_1_, Vitamin B_2_, Vitamin B_6_, Vitamin B_12_, Niacin, Pantothenic Acid, Biotin, Choline, Inositol, Carnitine, Taurine, Potassium, Chloride, Sodium, Calcium, Phosphorus, Magnesium, Iron, Copper, Iodine, Selenium, Chromium, Molybdenum, Zinc, Manganese
Gluten Free Lactose clinically irrelevant

Legend. DSF = Diabetes-Specific Formula; EN= Enteral Nutrition; Kcal = Kilocalories; Carb = carbohydrates.

**Table 2:** Variation in laboratory parameters in diabetic patients receiving DSF.

	Baseline (Mean)	6 Months (Mean)	Δ
BG (mg/dL)	145	128	−17
HbA1c (%)	9.8%	7.7%	−0.8
Creatinine (mg/dL)	0.98	0.9	−0.08
Uric Acid (mg/dL)	9.6	7.4	−2.2
TC (mg/dL)	288	240	−48
HDL (mg/dL)	31	42	+11
LDL (mg/dL)	187	120	−67
TG (mg/dL)	300	160	−140

Legend. DSF: Diabetes-Specific Formula; BG = Blood Glucose; HbA1c = Hemoglobin A1c; TC = Total Cholesterol; HDL = High-Density Lipoprotein; LDL = Low-Density Lipoprotein; TG = Triglycerides; Δ = mean difference.

## Data Availability

The data supporting this research are available upon request from the corresponding author for data protection reasons.
